# Ambient air pollution and adverse birth outcomes: a natural experiment study

**DOI:** 10.1186/s12963-015-0050-4

**Published:** 2015-07-18

**Authors:** Cheng Huang, Catherine Nichols, Yang Liu, Yunping Zhang, Xiaohong Liu, Suhong Gao, Zhiwen Li, Aiguo Ren

**Affiliations:** Milken Institute School of Public Health, George Washington University, Washington, DC USA; Rollins School of Public Health, Emory University, Atlanta, USA; Beijing Haidian Maternal and Child Health Hospital, Beijing, China; Institute of Reproductive & Child Health, School of Public Health, Peking University/Ministry of Health Key Laboratory of Reproductive Health, Beijing, China; Department of Global Health, Milken Institute School of Public Health, George Washington University, 950 New Hampshire Ave, office 407, Washington, DC 20052 USA

## Abstract

**Background:**

Radical regulations to improve air quality, including traffic control, were implemented prior to and during the 2008 Beijing Olympic Games. Consequently, ambient concentrations of nitrogen dioxide (NO_2_) and particular matter 10 micrometers or less (PM_10_), were reduced in a distinct and short window of time, which presented a natural experiment for testing the relationships between maternal exposure to PM_10_ and NO_2_ during pregnancy and adverse birth outcomes.

**Methods:**

We estimated the effect of PM_10_ and NO_2_ exposure during each trimester of gestation on the risk of preterm birth among live births and the birth weight among term babies. The data were based on 50,874 live births delivered between January 1, 2006 and December 31, 2010 at the Beijing Haidian Maternal and Child Health Hospital. Air monitoring data for the same period were obtained from the Beijing Municipal Environmental Monitoring Center.

**Results:**

Among full-term births, maternal exposure to NO_2_ in the third trimester predicted birth weight, with each 10-unit increment (per 10 ug/m^3^) in NO_2_ concentration associated with a 13.78 g (95 % confidence interval: −21.12, −6.43; p < 0.0001) reduction in birth weight. This association was maintained after adjusting for other pollutants, including carbon monoxide (CO), sulfur dioxide (SO_2_), and PM_10_. No relationship was found between the concentration of PM_10_ and low birth weight among full-term births. Neither PM_10_ nor NO_2_ concentrations predicted the risk of premature birth.

**Conclusions:**

Exposure to ambient air pollution during certain periods of pregnancy may decrease birth weight, but the effect size is small.

## Introduction

Adverse birth outcomes, including preterm birth and low birth weight, are an important global public health issue. Fifteen million babies are born premature (less than 37 weeks) worldwide each year [1]. Low birth weight (less than 2500 grams at birth) affects more than 20 million infants worldwide, representing 15 % of all births [2].

Experimental studies on laboratory animals found changes in reproductive function after the animals were exposed to air pollution [3–6]. In humans, increasing evidence suggests that air pollution plays a role in adverse birth outcomes [7], however, the strength of the evidence varies depending on the air pollutants and outcomes examined [8, 9]. In particular, many existing studies are subject to methodological shortcomings [7]. For example, simply linking birth outcomes of residents in different geographic areas with residence-based measurements of maternal exposure to ambient air pollution during pregnancy is problematic [10]. Residential areas close to industrial zones and highways typically have more affordable housing but are generally more polluted. Residents in these areas are also usually at a lower socio-economic status (SES). They may be less aware of environmental health hazards and therefore might invest less in personal and family health care. Such confounding factors, when uncontrolled, may bias the estimates of the health effects of ambient air pollution [6, 11]. Time-series regression studies that examine changes in air pollution levels driven by seasonality or meteorology with health outcomes usually face a dilemma: directly controlling for seasonality in the analysis may result in collinearity problems, because seasonality is highly associated with air pollutant concentrations. Additionally, controlling for proxies of seasonality, such as food availability, may miss important confounding factors such as seasonal patterns of physical exercise that are not measured or observed [6–8].

**Fig. 1:**
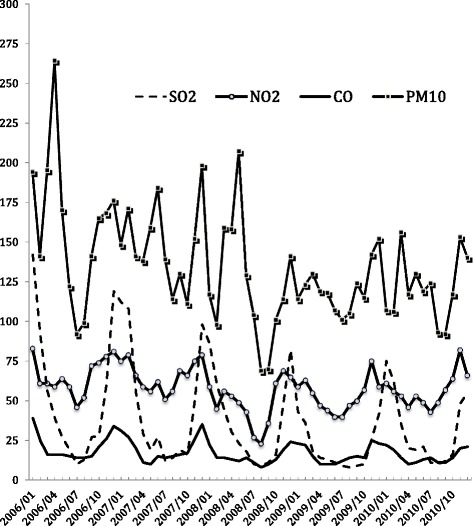
Concentration of ambient pollutants in Beijing during 2006–2010. Note: Pollutants concentrations exhibited at ug/m^3^ scales for SO_2_, NO_2_, PM_10_ and a 0.1 mg/m^3^ scale for CO

**Fig. 2:**
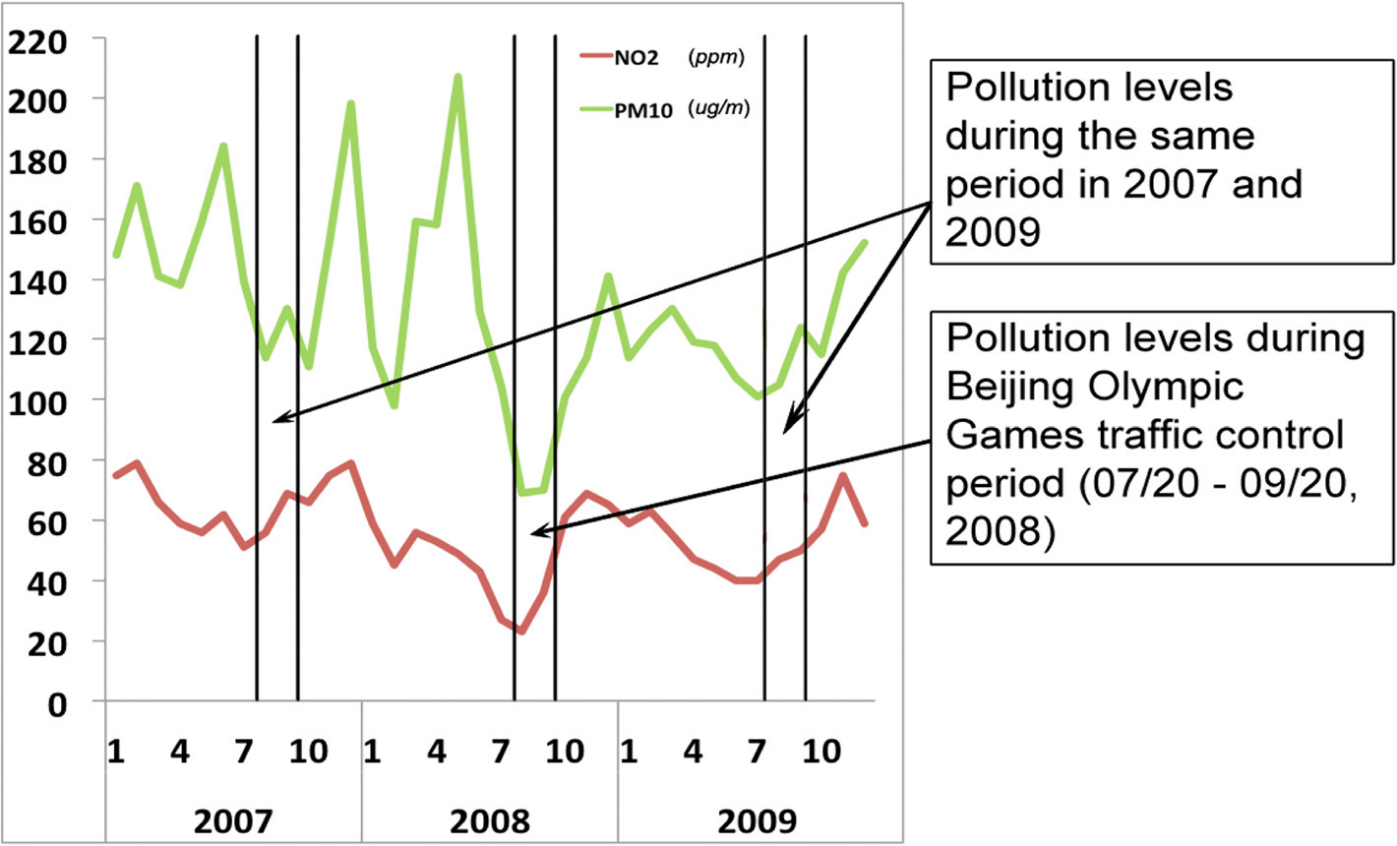
NO_2_ and PM_10_ concentrations before, during, and after the Beijing Olympic Games Traffic Control period

In the absence of randomized controlled trials on humans due to ethical concerns, these methodological limitations can be addressed with a natural experiment design that examines changes in air pollution levels caused by external forces, such as environmental regulations, which are usually less subject to spatial and temporal confounding factors [6, 7, 10, 12]. The 2008 Beijing Olympic Games present an opportunity to conduct a natural experiment. Beijing, one of the largest metropolises in the world with a population of over 15 million and more than 3 million vehicles, has been heavily polluted [13, 14]. The concentrations of ambient pollutants maintain high levels in Beijing, especially in the winter (Fig. 1). In an attempt to host the Olympic Games (August 8–24, 2008) and the subsequent Paralympic Games (September 6–17, 2008) with acceptable air conditions, the Chinese Central Government implemented regulations and measures to reduce pollution levels known during the Olympic Games Traffic Control period (July 20-September 20, 2008). The government’s tactics included closing down factories with emissions and halting construction projects in Beijing and surrounding regions. The government also enforced an alternate-day driving policy that led to a significant reduction in the concentration of ambient air pollutants, in particular PM_10_ and NO_2_, which are primarily generated by motor vehicles in these areas [14, 15]. For example, the average concentration of PM_10_ in Beijing was reduced from 130 ug/m^3^ in July-September 2007 to approximately 70 ug/m^3^ during the Olympic Games Traffic Control period in 2008 (Fig. 2). Such substantial reductions in NO_2_ and PM_10_ and the resurgence in air pollution after the Games, when these temporary restrictions were lifted, generated a clear and relatively brief window of non-exposure, making the Games an ideal natural experiment for studying the health effects of ambient air pollutants.

In this study, we utilized the 2008 Beijing Olympic Games as a natural experiment to examine the effects of ambient air pollution on adverse birth outcomes and to investigate which trimester of gestation was most susceptible to these exposures in relation to preterm birth and low birth weight outcomes.

## Materials and methods

### Data and measurement

#### Ambient air pollution

We used data on ambient air pollutants from 28 air quality monitoring sites located in the metropolitan area of Beijing, which are operated by the Beijing Municipal Environmental Monitoring Center, an institutional agency affiliated with the Beijing Municipal Environmental Protection Bureau. The Chinese government monitors four air pollutants considered hazardous to human health: sulphur dioxide (SO_2_), carbon monoxide (CO), nitrogen dioxide (NO_2_), and particulate matter 10 micrometers or less (PM_10_). At each site, a tapered element oscillating microbalance (TEOM) (model 1400a, Rupprecht and Patashnick, now Thermo Scientific) measured the PM_10_ concentrations, a pulsed fluorescence gas analyzer (TE43C, Thermo Scientific) recorded SO_2_ concentrations, a chemiluminescence technology device (TE42C, Thermo Scientific) measured NO_2_ concentrations, and a gas filter correlation analyzer (TE48C, Thermo Scientific) recorded CO concentrations. All measurements were recorded hourly. They were averaged to daily levels at all of the sites and then combined and averaged to obtain citywide daily levels. The arithmetic mean of the citywide daily levels was then calculated to obtain citywide monthly level.

#### Study sample and measurement

The data of maternal and child health were obtained from the Beijing Haidian Maternal & Child Health Hospital (HMCHH), which provides maternal and child health care to Beijing residents, primarily those in the Haidian district. In addition, the HMCHH performs medical exams on approximately 10,000 newborns annually. Data on birth date, education, household registration (*Huji)* status, and fertility history of women were collected at admission. Birth weight was measured within 24 h after delivery. Gestational age (GA) was determined from the date of the mother’s last menstrual period confirmed by a sonographic examination prior to 20 weeks of gestation. The quality of the data has been discussed elsewhere and is considered high-standard. [16] We included all newborns (*n* = 50,874) delivered at HMCHH between January 1, 2007, and December 31, 2010, as well as births delivered in late 2006, when the hospital began collecting information on all of the variables included in this study. This relatively short time frame helps to mitigate potential confounding bias associated with long-term changes, such as improvements in prenatal health care and nutritional transitions. We used the standard definitions of preterm birth, which is a baby born at less than 37 weeks gestational age, and trimester divisions of 0–13 weeks, 14–26 weeks, and 27 weeks to birth. We assigned each newborn to a corresponding daily exposure for each pollutant, starting from the date of conception until the date of birth, which was then averaged for each trimester of gestation.

### Statistical analysis

We applied multi-pollutant models to investigate the effects of exposure to PM_10_ and NO_2_, two pollutants that exhibited significant reductions in concentrations due to temporary regulations during the Beijing Olympic Games, and we quantified such effects on each birth outcome by trimester of gestation. Similar analytical strategy has been presented elsewhere [11]. The model, of which the main outcome is birth weight of term children, is expressed as:

$$ \begin{array}{l} Outcom{e}_{\mathit{\mathsf{i}}y\mathit{\mathsf{m}}}={\displaystyle \sum_{t=1}^3\Big(P{M_{10}}^t{\beta_{\left(p{m}_{10}\right)}}^t}+N{O_2}^t{\beta_{\left(N{O}_2\right)}}^t+{Temp}^t\Big)\\ {}+V{X}_{iym}+{\varepsilon}_{iym}\end{array} $$

*Outcome*_*iym*_ refers to the birth weight of infant *i* who was born in the year *y* and the month *m*. Ambient air pollution levels in the first, second, and third trimester of the pregnancy was denoted by *t*. For example, *PM*_10_^1^ refers to concentration of PM_10_ in the first trimester and *NO*_2_^2^ refers to concentration of NO_2_ in the second trimester. VX stands for a variety of the infants’ and parents’ characteristics measured in the data, including sex of the infant, the household registration (*Huji*) of the family (urban *Huji* of Beijing, rural *Huji* of Beijing, urban *Huji* of other province, or rural *Huji* of other province), maternal age at delivery, and parity (one, or two and above live births). Because seasonality of conception is associated with both socio-economic factors and birth outcomes [6], we controlled for the seasonal effect by including dummy variables of month of conception. We also included daily precipitation, as well as daily minimum and maximum temperatures averaged over each trimester of gestation in the model (Temp), and we controlled for the SO_2_ and CO concentrations in the first, second, and third trimester of the pregnancy. In addition, to examine the crude effect of each individual pollutant, we produced a series of single-pollutant models for PM_10_ and NO_2._ Because missing data was rare, we excluded cases with missing values from the regression analysis. All analyses were performed with the software package SAS, version 8.2 (SAS Institute, Inc., Cary, North Carolina). This study was approved by the Institutional Review Board (IRB) at George Washington University.

## Results

Table 1 shows the average concentration and correlation coefficients of ambient air pollutants (SO_2_, NO_2_, CO, PM_10_) in Beijing from 2006 to 2010. The concentrations of SO_2_, NO_2_, CO, and PM_10_ were positively correlated with each other (0.45 < r < 0.87). The concentrations of all pollutants were far greater than the World Health Organization (WHO) air quality standard. For example, the annual mean of PM_10_ in Beijing during the period 2006–2010 is 134.72 ug/m^3^, about six times higher than recommended by the WHO (20 ug/m^3^) [17].

**Table 1:** Characteristics and correlations of monthly average concentration of ambient pollutants in Beijing, 2006-2010

Pollutants	Concentration				Correlation coefficients				
	Mean	Std dev	Min	Max		SO_2_	NO_2_	CO	PM_10_
SO_2_ (ug/m^3^)	37.52	32.03	8.00	142.00	SO_2_	1.00	0.64	0.87	0.45
NO_2_ (ug/m^3^)	58.07	13.10	23.00	83.00	NO_2_	0.64	1.00	0.78	0.55
CO (mg/m^3^)	1.73	0.68	0.80	3.90	CO	0.87	0.78	1.00	0.45
PM_10_ (ug/m^3^)	134.72	35.16	69.00	264.00	PM_10_	0.45	0.55	0.45	1.00

Results from descriptive analysis show that 6.3 % of the newborns delivered at HMCHH were born prematurely (GA less than 37 complete weeks). 54.7 % of the infants were born to parents whose household registration (*huji*) was registered as “Beijing Urban (*Cheng Qu*)”, while 27.2 % of the infants were born to parents registered as “urban *huji* of other province”. The average maternal age at delivery was 29.2 years with a standard deviation of 3.6. As a result of the strictly enforced family planning policy in urban China, the majority (87.9 %) of the newborns were firstborns.

Table 2 further displays the characteristics of the newborns and relevant maternal characteristics by both the sex of the newborns and their gestational status. The mean birth weight of the preterm sample, which was 2581.4 g for males (*n* = 1784) and 2477.2 g for females (*n* = 1387), was less than the mean birth weight of the term sample 3453.7 g for males (*n* = 24,842) and 3331.4 g for females (*n* = 22,600).

**Table 2:** Characteristics of mothers and newborns delivered at Beijing Haidian Maternal and Child Health Hospital, 2006–2010

Characteristics	Term sample (*N* = 47,671)		Preterm sample (*N* = 3,203)	
	Male (*N* = 24,843)	Female (*N* = 22,600)	Male (*N* = 1,784)	Female (*N* = 1,387)
Birth weight (grams)	3453.7	3331.4	2581.4	2477.2
(Std dev)	(415.1)	(402.8)	(523.9)	(513.6)
Birth year %				
*2006*	6.63	6.25	6.00	6.49
*2007*	23.81	23.99	21.69	23.22
*2008*	23.60	23.81	27.19	24.44
*2009*	24.97	24.62	26.23	25.67
*2010*	21.00	21.33	18.89	20.19
Household registration (*Huji*) %				
*Urban Huji of Beijing*	54.82	55.89	48.71	49.53
*Rural Huji of Beijing*	1.82	1.84	1.85	1.95
*Other province, urban*	27.33	27.44	26.51	26.03
*Other state, rural*	15.91	14.73	22.65	22.42
*Missing*	0.12	0.11	0.28	0.07
Maternal age at delivery (years)				
Mean (STD)	29.2 (3.7)	29.2 (3.6)	29.3 (4.2)	29.3 (4.3)
Parity %				
*1*	87.58	89.99	80.55	84.21
*2+*	12.41	9.98	19.34	15.65
*Missing*	0.02	0.03	0.11	0.14

Table 3 presents the estimates of the correlations between the concentrations of ambient air pollutants and preterm birth. Results from both the single pollutant model without controlling for the concentration of any other pollutant and the multi-pollutant model controlling for other pollutants suggest that neither the concentration of NO_2_ nor PM_10_ predicted the risk of preterm delivery.

**Table 3:** Effects of concentrations of ambient air pollutants on preterm delivery

Concentration of ambient air pollutant	NO_2_ (per 10 ug/m^3^)				PM_10_ (per 10 ug/m3)			
	Single pollutant model		Multi-pollutant model		Single pollutant model		Multi-pollutant model	
	OR (95 % CI)	*P*-value	AOR (95 % CI)	*P*-value	OR (95 % CI)	*P*-value	AOR (95 % CI)	*P*-value
1^st^ trimester	0.98 (0.89, 1.08)	0.732	0.93 (0.70, 1.25)	0.645	0.98 (0.94, 1.02)	0.258	1.00 (0.92, 1.08)	0.992
2^nd^ trimester	0.94 (0.87, 1.02)	0.141	0.22 (0.68, 1.08)	0.194	0.96 (0.91, 1.00)	0.074	0.97 (0.88, 1.08)	0.597
3^rd^ trimester	1.01 (0.89, 1.15)	0.897	0.96 (0.72, 1.29)	0.805	0.98 (0.94, 1.02)	0.299	1.01 (0.90, 1.12)	0.923

*OR* odds ratio, *AOR* adjusted odds ratio

Table 4 presents the correlations of concentrations of ambient pollutants and birth weight among infants born full term. Results from the single pollutant model suggest that without controlling for the concentration of other pollutants, a higher concentration of NO_2_ in the third trimester predicted lower birth weights, with each 10-unit increment (per 10 ug/m^3^) in NO_2_ concentration associated with a 13.78 g (95 % confidence interval [CI]: −21.12, −6.43; p < 0.001) reduction in birth weight. Results from the multi-pollutant model suggest that this association is maintained after adjusting for other pollutants including CO, SO_2_, and PM_10_, and that each ten-unit increment (per 10 ug/m^3^) in NO_2_ concentration is associated with a 14.78 g (95 % CI: −29.13, −0.43; *p* = 0.044) reduction in birth weight. These effects are statistically significant but considered small as the effect sizes are less than 0.1 [18]. No relationship was found between a concentration of PM_10_ and birth weight, with or without adjustment for the concentration of other pollutants.

**Table 4:** Effects of concentration of ambient air pollutants on birth weight of infants born full term

Concentration of ambient air pollutant	NO_2_ (per 10 ug/m^3^)				PM_10_ (per 10 ug/m3)			
	Single pollutant model		Multi pollutant model		Single pollutant model		Multi pollutant model	
	Change of birth weight	*P*-value	Change of birth weight	*P*-value	Change of birth weight	*P*-value	Change of birth weight	*P*-value
1^st^ trimester	5.85 (−1.03, 12.74)	0.096	−1.43 (−17.16, 14.3)	0.859	−1.18 (−3.21, 0.85)	0.254	−1.63 (−5.66, 2.40)	0.427
2^nd^ trimester	−3.72 (−11.76, 4.32)	0.364	−9.74 (−28.85, 9.38)	0.318	0.43 (−1.74, 2.60)	0.699	2.54 (−3.57, 8.66)	0.415
3^rd^ trimester	−13.78 (−21.12, −6.43)	0.000	−14.78 (−29.13, −0.43)	0.044	−2.55 (−5.29, 0.20)	0.069	4.67 (−2.76, 12.10)	0.218

## Discussion

The 2008 Beijing Olympic Games provided a natural experiment setting in which the strict environmental regulations implemented by the Chinese government resulted in a substantial but temporary improvement in air quality. This enabled us to examine the effects of ambient air pollution exposure on adverse birth outcomes. We did not find any correlations between maternal exposure to ambient pollutants and preterm births. However, we observed small-magnitude associations between maternal exposure to NO_2_ in the third trimester and birth weight. Specifically, maternal exposure to NO_2_ in the third trimester predicted a reduced birth weight among full-term newborns, with or without controlling for other pollutants.

Several studies have examined fetal growth and exposure to ambient air pollution by trimester, and the findings have been widely varied, making conclusions difficult to draw [7]. Some studies have found an association between fetal growth and first-trimester exposure to PM_10_ [19–21]. However, another study found an association with second-trimester exposure [22], while a final study found an association with third-trimester exposure [23]. The evidence regarding the effect of exposure to ambient air pollution on fetal growth for other pollutants is similarly mixed. For example, several studies found an association between fetal growth and first-trimester exposure to CO [21, 23–26], while others found an association during the third trimester [23, 26]. Further research on air pollution and birth outcomes by exposure window is needed.

Although not yet validated, several potential mechanisms may explain the significant, albeit small, relationship between ambient air pollution exposure during pregnancy and low birth weight. [9] Ambient air pollutants may induce oxidative stress and inflammation, as well as alter blood coagulation and hemodynamic responses for the fetus and placenta [27]. Such changes could affect nutrient intake by the fetus, potentially impairing fetal growth [27]. In non-pregnant individuals, exposure to ambient pollutants has also been associated with endothelial function and plasma viscosity [28]. Researchers have hypothesized that such alterations in artery vascoconstriction could affect maternal-placental exchanges and thus affect fetal growth [6]. A fetus’ vulnerability to environmental toxicants may also result in the abnormal development of organ systems during this critical window of growth [29]. Further research of these effects on laboratory animals and studies on pregnant women using biomarkers may provide further insight on the biological mechanisms [6].

The findings from this study should be interpreted with caution. First, this study extrapolated information from city-wide measurements of ambient air pollution, and we were unable to draw conclusions based on exposures to air pollutants at the individual level. Certain uncontrolled factors, such as occupation and mobility, may have affected each individual’s level of exposure to ambient air pollutants. Second, our natural experiment design may have been subject to other changes that simultaneously contributed to the improvement of air quality during the Olympic Games. For example, exposure to secondhand smoking during pregnancy, a well-known risk factor of preterm birth and low birth weight of newborns, may have been mitigated during the games when public smoking regulations were likely implemented, decreasing the risk of adverse birth outcomes. Future studies may benefit from a prospective cohort research design that allows researchers to use biomarkers of exposure at an individual level and the collection of detailed information on characteristics of study subjects, including behaviors, when feasible [6].

Nevertheless, our study, based on a natural experiment approach, provided further evidence that ambient air pollution may be associated with reduced birth weights, albeit the effect sizes are small, which is consistent with previous studies based on other designs [6, 7]. Such small effects may not be clinically important, but may be consequential at the population level in areas where there is elevated air pollution, including parts of China. In China’s mega cities, on 10 to 30 % of days, the concentration of air pollutants exceeds the Chinese National Ambient Air Quality Standard (CNAAQS) of Grade-II [30]. Evidence compiled from previous studies, including those conducted on the United States Clean Air Act, has suggested that strict regulatory interventions lowering air pollution levels can substantially improve population health, especially among vulnerable populations including pregnant women and infants [10–12, 31].

## References

[1] World Health Organization. Preterm birth 2013: [http://www.who.int/mediacentre/factsheets/fs363/en/ (accessed Sep 15, 2014) pp.].

[2] UNICEF. Undernourishment in the womb can lead to diminished potential and predispose infants to early death 2014: [http://data.unicef.org/nutrition/low-birthweight (accessed Sep 15, 2014) pp.].

[3] Archibong AE, Inyang F, Ramesh A, Greenwood M, Nayyar T, Kopsombut P (2002). Alteration of pregnancy related hormones and fetal survival in F-344 rats exposed by inhalation to benzo(a)pyrene. Reprod Toxicol.

[4] Mohallem SV, de Araujo Lobo DJ, Pesquero CR, Assuncao JV, de Andre PA, Saldiva PH (2005). Decreased fertility in mice exposed to environmental air pollution in the city of Sao Paulo. Environ Res.

[5] Rocha ESIR, Lichtenfels AJ, Amador Pereira LA, Saldiva PH (2008). Effects of ambient levels of air pollution generated by traffic on birth and placental weights in mice. Fertil Steril.

[6] Slama R, Darrow L, Parker J, Woodruff TJ, Strickland M, Nieuwenhuijsen M (2008). Meeting report: atmospheric pollution and human reproduction. Environ Health Perspect.

[7] Woodruff TJ, Parker JD, Darrow LA, Slama R, Bell ML, Choi H (2009). Methodological issues in studies of air pollution and reproductive health. Environ Res.

[8] Sram RJ, Binkova B, Dejmek J, Bobak M (2005). Ambient air pollution and pregnancy outcomes: a review of the literature. Environ Health Perspect.

[9] Stieb DM, Chen L, Eshoul M, Judek S (2012). Ambient air pollution, birth weight and preterm birth: a systematic review and meta-analysis. Environ Res.

[10] Currie J, Walker R (2011). Traffic Congestion and Infant Health: Evidence from E-ZPass. Am Econ J-Appl Econ.

[11] Currie J, Neidell M, Schmieder JF (2009). Air pollution and infant health: Lessons from New Jersey. J Health Econ.

[12] Currie J, Ray SH, Neidell M (2011). Quasi-experimental studies suggest that lowering air pollution levels benefits infants' and children's health. Health Aff (Millwood).

[13] Hao J, Wang L (2005). Improving urban air quality in China: Beijing case study. J Air Waste Manag Assoc.

[14] Wang Y, Hao J, McElroy MB, Munger JW, Ma H, Chen D (2009). Ozone air quality during the 2008 Beijing Olympics: effectiveness of emission restrictions. Atmos Chem Phys.

[15] United Nations Environment Programme (2009). Independent Environmental Assessment: Beijing 2008 Olympic Games.

[16] Zhang YP, Liu XH, Gao SH, Wang JM, Gu YS, Zhang JY (2012). Risk factors for preterm birth in five Maternal and Child Health hospitals in Beijing. PLoS One.

[17] World Health Organization (2006). Air quality guidelines. Global update 2005. Particulate matter, ozone, nitrogen dioxide and sulfur dioxide.

[18] Cohen J (1992). A power primer. Psychol Bull.

[19] Dugandzic R, Dodds L, Stieb D, Smith-Doiron M (2006). The association between low level exposures to ambient air pollution and term low birth weight: a retrospective cohort study. Environ Health.

[20] Hansen C, Neller A, Williams G, Simpson R (2006). Maternal exposure to low levels of ambient air pollution and preterm birth in Brisbane, Australia. BJOG.

[21] Medeiros A, Gouveia N (2005). Relationship between low birthweight and air pollution in the city of Sao Paulo, Brazil. Rev Saude Publica.

[22] Mannes T, Jalaludin B, Morgan G, Lincoln D, Sheppeard V, Corbett S (2005). Impact of ambient air pollution on birth weight in Sydney, Australia. Occup Environ Med.

[23] Bell ML, Ebisu K, Belanger K (2007). Ambient air pollution and low birth weight in Connecticut and Massachusetts. Environ Health Perspect.

[24] Gouveia N, Bremner SA, Novaes HM (2004). Association between ambient air pollution and birth weight in Sao Paulo, Brazil. J Epidemiol Community Health.

[25] Salam MT, Millstein J, Li YF, Lurmann FW, Margolis HG, Gilliland FD (2005). Birth outcomes and prenatal exposure to ozone, carbon monoxide, and particulate matter: results from the Children's Health Study. Environ Health Perspect.

[26] Wilhelm M, Ritz B (2005). Local variations in CO and particulate air pollution and adverse birth outcomes in Los Angeles County, California, USA. Environ Health Perspect.

[27] Kannan S, Misra DP, Dvonch JT, Krishnakumar A (2007). Exposures to airborne particulate matter and adverse perinatal outcomes: a biologically plausible mechanistic framework for exploring potential. Cien Saude Colet.

[28] Pope CA, Dockery DW (2006). Health effects of fine particulate air pollution: lines that connect. J Air Waste Manag Assoc.

[29] Calabrese EJ (1986). Age and Susceptibility to Toxic Substances.

[30] Chan CK, Yao X (2008). Air pollution in mega cities in China. Atmos Environ.

[31] U.S. Environmental Protection Agency Office of Air and Radiation. The Benefits and Costs of the Clean Air Act from 1990 to 2020: Final Report – Rev. A2011: [http://www.epa.gov/air/sect812/feb11/fullreport_rev_a.pdf (accessed Sep 15, 2014) pp.].

